# Involvement of Surface Receptors in the Uptake and Cellular Responses Induced by Cationic Polyamine-Based Carbon Dots in Macrophages

**DOI:** 10.3390/toxics13090731

**Published:** 2025-08-30

**Authors:** Agathe Cerland, Ezeddine Harmouch, Mickaël Rapp, Luc Lebeau, Françoise Pons, Carole Ronzani

**Affiliations:** Laboratoire de Chémo-Biologie Synthétique et Thérapeutique, UMR 7199, Faculté de Pharmacie, CNRS, Université de Strasbourg, 67400 Illkirch, Francellebeau@unistra.fr (L.L.);

**Keywords:** carbon dots, carbon nanoparticles, macrophages, scavenger receptors, SR-A1, MERTK signaling pathway, protein corona, nanotoxicology, immune response

## Abstract

Cationic polyamine-based carbon dots (CDs) are increasingly being explored for biomedical applications. These ultrasmall (<10 nm) fluorescent nanoparticles, synthesized from organic precursors and functionalized with polyamines, possess a strong positive surface charge that enables efficient complexation and delivery of nucleic acids, making them promising candidates for gene therapy. However, the mechanisms by which the immune system, particularly macrophages, recognizes and responds to these nanomaterials remain poorly understood. In this study, we investigated the role of surface receptors in the uptake and biological effects of cationic polyamine-based CDs in macrophages. Our data showed that Fc receptors and the Toll-like receptor 4 (TLR4) were minimally involved in CD internalization and associated cellular responses in contrast to scavenger receptors (SRs). Indeed, SR inhibition reduced CD-induced cell viability loss, LDH release, and secretion of the pro-inflammatory cytokine IL-1β. Among SRs, SR-A1 was identified as a key receptor mediating CD recognition and toxicity, likely through activation of the MERTK signaling pathway. Importantly, these mechanisms occurred in the absence of serum, indicating that protein corona formation is not required for CD interaction with macrophage surface receptors. Overall, our findings highlight the prominent role of SRs, particularly SR-A1, as receptors recognizing cationic polyamine-based CDs on the surface of macrophages, and provide new insights into the cellular mechanisms underlying the immunotoxicity of these carbon-based nanomaterials.

## 1. Introduction

Over the last ten years, carbon-based nanoparticles (NPs), commonly referred to as carbon dots (CDs), have gained attention as highly promising nanomaterials with broad application potential [1,2]. CDs are quasi-spherical NPs that exhibit unique features, including an ultra-small dimension (<10 nm), strong chemical stability, excellent water dispersibility, tunable intrinsic fluorescence, and notable resistance to photobleaching [3]. They are most often prepared by carbonizing inexpensive organic precursors (e.g., citric acid, glucose) in the presence of catalysts and/or passivating agents. The synthesized CDs can be readily modified through chemical functionalization, leading to nano-objects enriched with various surface chemical groups [4,5]. Beyond their utilization in advanced industrial technologies, including optoelectronics, photovoltaics, and energy storage, CDs are increasingly investigated in the biomedical field, where they serve as versatile and efficient nanoplatforms for drug delivery, bioimaging, and theranostics [6,7,8]. In particular, cationic polyamine-based CDs are being actively developed as nanocarriers for gene therapy. Thanks to their positive surface charge, these CDs can complex nucleic acids into stable nanoassemblies, thereby enabling efficient transfection [9]. However, to safely harness CDs in nanomedicine, a more thorough understanding of their interactions with biological systems is essential.

With the highlighting of the potential immunological effects of NPs [10,11,12], taking into account the interactions of nanomaterials with the immune system appears crucial in the development of new nanotechnologies for health. Although carbon is generally regarded as a non-toxic element, several carbon-based nanomaterials, such as carbon nanotubes, carbon black, graphene, or fullerenes, have raised safety concerns towards immune cells, notably due to their ability to induce oxidative stress, inflammatory response, and cell death [13,14,15,16]. Initially considered as being biocompatible [1], increasing evidence suggests that CDs may also induce toxicological effects, depending on their physicochemical characteristics [17,18,19]. In particular, surface charge has emerged as a major determinant of CD-induced toxicity. Using a large library of CDs differing in size, surface charge, and surface chemistry, our group demonstrated that anionic CDs were consistently non-toxic, whereas cationic polyamine-based CDs, especially those with a marked surface charge density, triggered cytotoxicity in cultured human macrophages and inflammation in the lungs of mice [20,21]. At the mechanistic level, we showed that cationic polyamine-based CDs are rapidly internalized by macrophages, accumulate in lysosomes, and cause lysosomal destabilization, leading to mitochondrial dysfunction, oxidative stress, and activation of the NLRP3 inflammasome [22]. This cascade ultimately drives a pro-inflammatory response and pyroptotic cell death [23]. Based on these data, we proposed an adverse outcome pathway (AOP) in which uptake of cationic polyamine-based CDs by macrophages constitutes the molecular initiating event leading to lung inflammation [24]. However, how cationic polyamine-based CDs are recognized by macrophages for their internalization remains unknown.

Macrophages express a broad spectrum of membrane receptors known as pattern-associated recognition receptors (PRRs), which are able to detect molecular signatures, whether associated with pathogens (namely, PAMPs for pathogen-associated molecular patterns) or cellular damages (namely, DAMPs for damage-associated molecular patterns) [25]. Recently, it has been proposed that NP-associated molecular patterns (NAMPs) may constitute a new type of danger signal that could trigger NP recognition by phagocytic cell surface receptors and subsequent immunological signaling cascades [11,26]. Initially identified to bind and remove modified lipoproteins, the scavenger receptor (SR) superfamily has been proposed to be implicated in the recognition, internalization, and inflammatory or cytotoxic responses of NPs [27]. Within this family, class-A scavenger receptors (SR-A), in particular SR-A1, have been shown to participate in macrophage responses to different NPs, such as silica [28], polystyrene [29] or gold NPs [30], or carbon nanotubes [31]. Although class-B SRs (SR-B) appear to play a predominant role in epithelial and endothelial cells rather than macrophages, several studies indicate that SR-B1 contributes to inflammasome activation and subsequent pro-inflammatory signaling in macrophages exposed to silica NPs [32]. Among other key macrophage surface receptors, Toll-like receptors (TLR) have also been shown to interact with NPs. For example, TLR4 was reported to bind cationic liposomes [33,34] or peptide-gold NPs [35], leading to a pro-inflammatory response [33]. Macrophages can also recognize NPs following protein corona formation, a dynamic process where proteins, including opsonins, adsorb onto the NP surface upon contact with biological fluids [36]. This opsonization process may influence NP cellular uptake and cytotoxicity [37,38]. In the same way as endogenous particles like monosodium urate or cholesterol crystals, opsonized manufactured NPs could be detected through complement or Fc receptors [27]. However, the mechanisms underlying the recognition of most NPs by macrophages remain poorly understood. Importantly, according to current knowledge, no report has specifically investigated the interactions between macrophage surface receptors and CDs, and particularly cationic polyamine-based CDs.

In this context, the objective of our study was to investigate the involvement of macrophage surface receptors in the recognition and cellular responses to cationic polyamine-based CDs. Our investigations focused on three major receptor families: the SR (especially SR-A1), the TLR (TLR4), and the Fc receptor (FcγRI). The experiments were conducted on phorbol 12-myristate 13-acetate (PMA)-differentiated THP-1 cells, as a well-established in vitro model for studying macrophage functions [39] and surface receptor-mediated interactions with NPs [40,41,42,43]. At first, we characterized the expression of some SRs, TLR4, and FcγRI on the surface of these macrophages. Subsequently, we explored the involvement of these receptors in the internalization and cellular effects of cationic polyamine-based CDs. At last, we examined the role of the protein corona in mediating CD recognition by macrophages.

## 2. Materials and Methods

### 2.1. Synthesis and Characterization of CDs

The cationic polyamine-based CDs analyzed in this study were synthesized and characterized following previously established protocols [22,38]. In short, citric acid (2.0 g), bPEI600 (8.0 g), and ultrapure water (50 mL) were combined in a beaker and heated at 160–170 °C with constant stirring. The solid residue obtained was dissolved in 0.1 N HCl, subjected to dialysis against 0.1 N HCl for 24 h, and then against ultrapure water for an additional 24 h. The dialyzed solution was filtered through a 0.22 μm PES membrane and subsequently freeze-dried, yielding 1.6 g of CDs in the form of an orange hygroscopic powder.

For characterization, fresh CD suspensions (1.0 mg/mL in 1.5 mM NaCl, pH 7.4) were used. The hydrodynamic diameter, polydispersity index (PdI), charge (zeta-potential, ζ-potential), and surface charge density were determined by dynamic light scattering (DLS) on a Zetasizer Nano ZS apparatus (Malvern Instruments, Palaiseau, France). Measurements of particle size, size distribution, and charge were performed in triplicate at 25 °C, and results were expressed as mean ± SD. Surface charge density was calculated from the variation of ζ-potential of CD suspensions upon titration with poly (acrylic acid) (PAA, MW ≈ 1800 Da, 1.5 mM NaCl, pH 7.4), as reported previously [24], and expressed in μmol/mg. The morphology of CDs was examined by transmission electron microscopy (TEM) using an LVEM5 microscope (Delong Instruments, Brno, Czech Republic) operated at 5 kV. For sample preparation, 0.5 μL of a CD suspension (1.0 mg/mL) was applied onto carbon-coated copper grids (Cu-300HD, Pacific Grid Tech, San Francisco, CA, USA) previously treated by glow discharge (90 V, 2 mA, 15 s). The grids were then allowed to dry at room temperature for a minimum of 2 h before imaging. Optical features of CDs were assessed by recording UV–visible and fluorescence spectra using a multimode reader (Varioskan Lux, Thermo Fisher Scientific, Illkirch-Graffenstaden, France).

### 2.2. Cell Culture

THP-1 cells (TIB-202™, LGC Standards, Molsheim, France) were maintained at 37 °C in a humidified 5% CO_2_ incubator using RPMI-1640 medium supplemented with L-glutamine (2 mM), 2-mercaptoethanol (0.05 mM), penicillin (100 U/mL), streptomycin (100 μg/mL), and 10% heat-inactivated FBS (all reagents from Life Technologies Limited, Paisley, UK). For experiments, cells were seeded into suitable culture plates (as described above) and differentiated into macrophages by adding 10 ng/mL PMA (Sigma-Aldrich, St Louis, MO, USA) overnight.

### 2.3. Characterization of the Macrophage Surface Receptors

Fluorescence Activated Cell Sorting (FACS) and confocal laser scanning microscopy (CLSM) were used to assess the presence of receptors at the surface of the THP-1-derived macrophages. For FACS, cells were seeded into 24-well plates at a density of 5 × 10^5^ cells per well and differentiated for 24 h, as indicated above. On the day of analysis, macrophages were rinsed with PBS, detached by trypsinization, washed in PBS containing 0.5% BSA, and incubated at room temperature for 10 min with a blocking solution (TruStain FcX™, BioLegend, London, UK). Cells were then labeled for 20 min with the following antibodies (all from BioLegend, London, UK): APC anti-human CD204 antibody (7C9C20 clone, 100 μg/mL), APC anti-human CD36 antibody (5271 clone, 100 μg/mL), APC anti-human CD284 antibody (HTA125 clone, 200 μg/mL), APC anti-human CD64 antibody (10.1 clone, 200 μg/mL), Alexa Fluor^®^ 700 anti-human CD11b antibody (ICRF44 clone, 200 μg/mL), and PE anti-human CD35 antibody (E11 clone, 25 μg/mL) to identify SR-A1, SR-B2, TLR4, Fc receptors, and CR3 and CR1 receptors, respectively. After staining, cells were washed once with PBS/BSA and twice with PBS before analysis on an LSRFortessa X 20™ cytometer (BD Biosciences, Le Pont de Claix, France). Fluorescence signals (30,000 events) were collected using APC (red laser), APC-Alexa 700 (red laser), or PE (yellow-green) channels, and data were processed with FlowJo™ (v10.3, Ashland, OR, USA). The results were expressed as the percentage of labeled cells.

For CLSM, THP-1 cells were plated into 8-well IbiTreat µ-Slides (1.5 polymer coverslip, IBIDI^®^, Ibidi GmbH, Gräfelfing, Germany) at 1 × 10^5^ cells per well and differentiated into macrophages. Following differentiation, cells were rinsed with PBS containing 0.5% BSA, then incubated for 1 h with APC-labeled antibodies against CD204, CD36, CD284, or CD64 to detect SR-A1, SR-B2, TLR4, or Fc receptors, respectively. After staining, samples were washed with PBS and incubated for 5 min with the MB488 probe (200 nM in PBS) to label cell membranes [44]. Microscopy was performed using a SP2 confocal microscope (Leica microsystems, Nanterre, France) with a 63× oil immersion objective (numerical aperture = 1.2). The antibodies and the MB488 probe were excited with 635 nm and 488 nm laser lines, respectively.

### 2.4. Macrophage Exposure to CDs and Pharmacological Inhibitors

THP-1 cells were seeded in 96-, 24-, or 6-well culture plates at a density of 1 × 10^5^, 5 × 10^5^, or 2 × 10^6^ cells per well, respectively, and differentiated into macrophages. The macrophages were then washed with PBS and pre-treated or not for 1 h at 37 °C with the SR inhibitors Fucoidan (10 or 50 µg/mL), Polyinosinic acid potassium salt (Poly-I, 10 or 50 µg/mL), or Polyguanylic acid potassium salt (Poly-G, 10, or 50 µg/mL), the TLR4 inhibitor Cli-095 (1 or 2 µg/mL); the FcR inhibitor Fc Block (2, 5, or 10 µL/well), and the MERTK inhibitor UNC569 (0.25, 0.5, 1, or 5 µM). Fucoidan, Poly-I, and Poly-G were from Sigma-Aldrich (Saint-Louis, MO, USA); Cli-095 was from InvivoGen (Toulouse, France); Fc Block from Biolegend (London, UK); and UNC569 was from (Sigma-Aldrich, St Louis, MO, USA). Then, the cells were exposed for 1 or 4 h to CD suspensions that were freshly prepared in culture medium at 12, 25, or 50 µg/mL, or to culture medium alone (controls). All cellular responses (CD cell uptake, cell viability, cell necrosis, IL-1β secretion, and MERTK or phospho-MERTK expression) were analyzed at the end of the CD incubation period. For studying the role of the protein corona in mediating the recognition of CDs by macrophages, some experiments were performed in the absence of serum. In this case, the CD preparation, the cell pre-treatment with receptor inhibitors, and the cell exposure to CDs were performed in serum-free culture medium. In all other experiments, and where not specified, all protocol steps were performed in culture medium containing 10% serum.

### 2.5. Assessment of CD Uptake by Macrophages

Macrophage uptake of CDs was analyzed by CLSM and FACS. For CLSM, THP-1 cells were seeded into 8-well IbiTreat µ-Slides at 1 × 10^5^ cells per well, differentiated, and incubated for 1 h with CDs (25 µg/mL) in either complete or serum-free medium. After incubation, cells were rinsed with serum-free medium, stained with MB488 (200 nM in PBS) to label membranes, and examined using a Leica SP2 microscope (63× oil immersion). CDs and MB488 were excited with 405 nm and 488 nm lasers, respectively.

For FACS, THP-1 cells were seeded into 24-well plates (5 × 10^5^ per well), differentiated, pre-treated or not with surface receptor inhibitors in complete or serum-free culture medium, and then incubated with 25 µg/mL CDs for 1 (serum-free culture medium) or 4 h (complete medium). Following exposure, cells were washed with PBS, detached by trypsinization, and further rinsed with PBS containing 0.5% BSA, then with PBS alone. Cell suspensions were analyzed on an LSRFortessa X 20™ cytometer (BD Biosciences, Le Pont de Claix, France). Fluorescence (30,000 events) was collected in the BV421 channel (violet laser). CD uptake was quantified as changes in mean fluorescence intensity (MFI) relative to untreated cells, and results were expressed as the ratio of MFI values. When studying the implication of macrophage surface receptors, results were expressed as percent of CD uptake inhibition.

### 2.6. Cell Viability and Necrosis Assays

Cell viability and necrosis were assessed with MTT and lactate dehydrogenase (LDH) assays, respectively. Cells were seeded in 96-well plates (1 × 10^5^ cells per well), differentiated into macrophages, pre-treated or not with surface receptor inhibitors in complete or serum-free culture medium, and then incubated with CDs (12, 25, or 50 µg/mL) for 4 h in complete or serum-free culture medium. After CD exposure, the cell supernatants were collected for the LDH or IL-1β assay, whereas 100 μL of MTT (1.0 mg/mL, in culture medium) was added to the cells. After 1 h incubation, MTT was removed, and cells were lysed with DMSO. Absorbance was read at 570 nm with background correction at 690 nm using a Varioskan LUX multimode reader (Thermo Fisher Scientific, Illkirch-Graffenstaden, France). Cell viability was expressed as the percentage absorbance of CD-treated cells relative to untreated cells. LDH release in supernatants was measured using the Cytotoxicity Detection Kit (Roche Diagnostics GmbH, Mannheim, Germany) according to manufacturer instructions, with results expressed as fold-change in the absorbance measured in the supernatants of CD-exposed cells relative to that measured in the supernatants of unexposed cells.

### 2.7. Interleukin-1β Assay

The secretion of Interleukin-1β (IL-1β) by macrophages was quantified using a sandwich ELISA (DuoSet ELISA kit, R&D Systems, Minneapolis, MN, USA). Briefly, 96-well high-binding microtiter plates were coated overnight at room temperature with 100 µL/well of capture antibody (4 µg/mL in PBS). Plates were then washed three times with PBS containing 0.05% Tween-20 and blocked for 1 h at room temperature with 300 µL/well of PBS containing 1% BSA. After additional washes, 100 µL of standards (0–250 pg/mL) or cell culture supernatants were added and incubated for 2 h at room temperature. Following washes, 100 µL/well of biotinylated detection antibody (200 ng/mL) was added and incubated for 2 h at room temperature. Plates were then washed and incubated with 100 µL/well of streptavidin-HRP (1:40 dilution) for 20 min at room temperature and in the dark. After final washes, 100 µL/well of TMB substrate was added, and the reaction was allowed to develop for 20 min in the dark before being stopped with 50 µL/well of 1 M H_2_SO_4_. Absorbance was read at 450 nm with correction at 570 nm using a microplate multimode reader (Varioskan LUX, Thermo Fisher Scientific, Illkirch-Graffenstaden, France). Cytokine concentrations were calculated from the standard curve and expressed as pg/mL.

### 2.8. Western Blotting Analysis

For western blot analysis, THP-1 cells were seeded into 6-well plates at a density of 2 × 10^6^ cells per well, differentiated into macrophages, pre-treated or not with a MERTK receptor inhibitor, and then incubated or not with CDs (25 µg/mL) for 4 h. After CD exposure, the cells were washed with PBS and lysed with the RIPA lysis buffer containing protease inhibitors (Pierce, Thermo Fisher Scientific, Illkirch-Graffenstaden, France). After determination of protein concentration by the BCA assay (Sigma-Aldrich, Saint-Louis, MO, USA), samples (10 µg of proteins) were denatured at 95 °C for 5 min in Laemmli buffer, loaded on a 4–20% polyacrylamide precast gel (Bio-Rad Laboratories, Marne La Coquette, France), run at 160 V, and transferred to a Hybond ECL^TM^ nitrocellulose membrane (GE Healthcare, London, UK). Blots were blocked with TBS-Tween (0.1%) containing 5% BSA for 1 h at room temperature and then incubated overnight at 4 °C with the following primary antibodies (all from Abcam, Amsterdam, Netherlands): anti-beta actin antibody (ab8227, 1:5000), anti-MERTK antibody (ab52968, 1:2000), and anti-phospho-MERTK antibody (ab14921, 1:1000). Blots were then washed four times for 10 min each using TBS-Tween. A secondary antibody (HRP-conjugated anti-rabbit IgG antibody, 1:10,000, GE Healthcare, London, UK) was then incubated for 1 h at room temperature. After three 10 min washes of the membranes with TBS-Tween, the protein bands were visualized with the Clarity ECL substrate (Bio-Rad Laboratories, Marne La Coquette, France), and the images were captured with an Amersham^™^ Imager 600 (GE Healthcare, London, UK). The band densities were analyzed using the ImageJ software (version 2.1).

### 2.9. Statistical Analysis

Data are presented as mean ± SEM from *n* = 2–6 biological replicates. Statistical comparisons were performed using Student’s *t*-test or one/two-way ANOVA with Dunnett’s or Sidak’s post hoc tests, via GraphPad Prism 6.0. The significance threshold was set at *p* < 0.05.

## 3. Results and Discussion

### 3.1. Physicochemical Characterization of CDs

**Figure 1:**
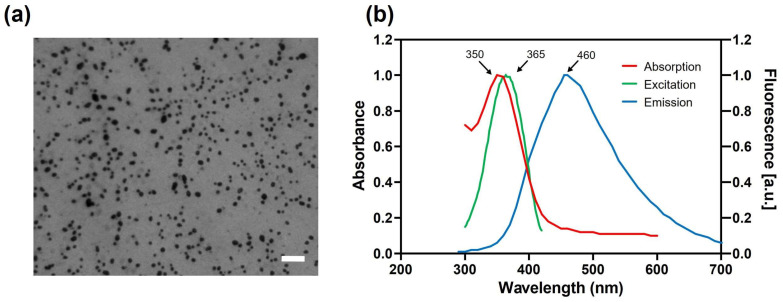
(**a**) Morphology of the cationic polyamine-based CDs as observed by TEM (scale bar = 200 nm). (**b**) Optical properties of the CDs: absorption (red), excitation (green), and emission (blue; excitation at 365 nm) spectra, scale bar = 20 µm.

The physicochemical characteristics of the studied CDs are summarized in Table 1. The cationic polyamine-based CDs exhibited a small hydrodynamic diameter. They had a cationic surface charge with a high charge density. TEM images revealed their rounded morphology (Figure 1a). The UV–vis absorption and fluorescence spectra were recorded (Figure 1b). The maximum absorption, fluorescence excitation, and fluorescence emission wavelengths were 350, 365, and 460 nm, respectively. These features confirm that studied the CDs are nano-sized cationic particles with intrinsic fluorescence, which allows their cell tracking without additional labeling. These expected characteristics are in agreement with previous reports [24].

### 3.2. Characterization of Surface Receptor Expression by THP-1-Derived Macrophages

**Figure 2:**
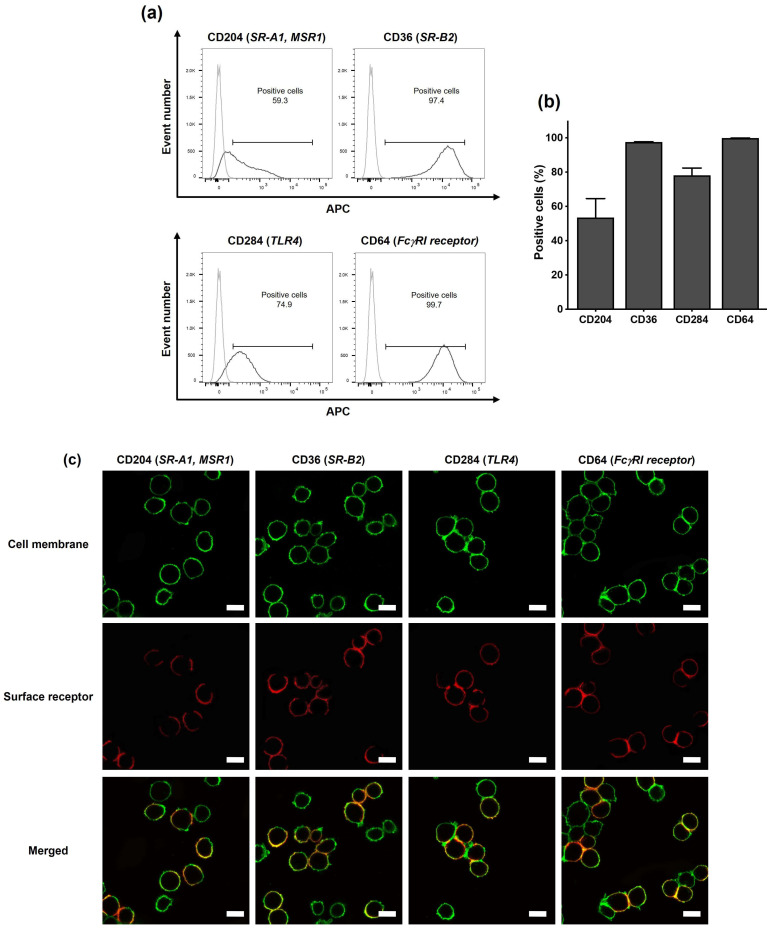
Determination of the presence of surface receptors on THP-1-derived macrophages by FACS and CLSM. (**a**) Staining and gating strategy to analyze the presence of the various receptors on the cell surface by FACS. (**b**) Quantification of the presence of the receptors on the cell surface by FACS. Results are expressed as a percentage of positive cells and are means ± SEM of *n* = 3 experiments. (**c**) Localization of the receptors at the cell surface, as evidenced by CLSM. Cell membranes are colored in green, and receptors appear in red (scale bar = 20 µm).

The first step in our study on the role of surface receptors in the recognition of cationic polyamine-based CDs by immune phagocytic cells was to characterize the presence of SR-A1 (CD204), SR-B2 (CD36), TLR4 (CD284), and FcγRI receptors (CD64) at the surface of THP-1-derived macrophages. To do this, PMA-differentiated THP-1 cells were labelled with antibodies specific to each receptor and analyzed by FACS or CLSM (Figure 2). FACS experiments showed that all the assessed receptors were present on THP-1-derived macrophages (Figure 2a), with a percentage of positive cells of 54% for CD204, 98% for CD36, 78% for CD284, and 99% for CD64 (Figure 2b). CLSM observations confirmed the presence of SR-A1, SR-B2, TLR4, and Fc receptors on the cell surface (Figure 2c), as evidenced by the presence of a labeling for each receptor (red label) that colocalized with the membrane labeling (green label). Thus, in accordance with the literature [45], we confirmed the presence of SRs, TLR4, and Fc receptors on the surface of THP-1-derived macrophages, making this model suitable for our study.

### 3.3. Involvement of Surface Receptors in CD Uptake and Toxicity

**Figure 3:**
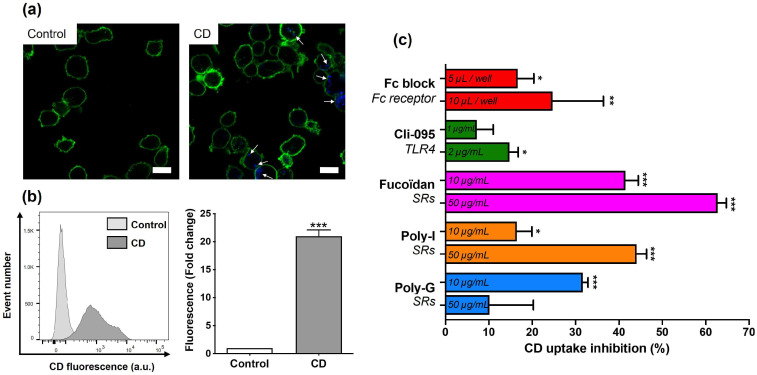
Involvement of surface receptors in cationic polyamine-based CD uptake by macrophages. (**a**) CD uptake in control cells (**left**) or cells exposed to 25 µg/mL of CDs (**right**) for 4 h, as assessed by CLSM. The cell membrane is colored in green, and CDs appear in blue and are indicated by white arrows (scale bar = 20 µm). (**b**) Quantification of CD uptake in cells exposed to CDs (25 µg/mL) for 4 h when compared to control cells, as assessed by FACS. Event number (**left**) and fold change in fluorescence intensity (**right**). Quantitative results are means ± SEM of *n* = 4 experiments. Statistical differences when compared to control were determined by Student’s *t*-test (*** *p* < 0.001). (**c**) Effect of surface receptor inhibition on CD uptake, as assessed by FACS. Cells were pre-treated with receptor inhibitors for 1 h before the addition of CDs (25 µg/mL), and CD internalization was measured at 4 h. Results were expressed as percent of inhibition of CD uptake and are means ± SEM of *n* = 4 experiments. Statistical differences were determined by ANOVA followed by the Dunnett’s test. * *p* < 0.05, ** *p* < 0.01, and *** *p* < 0.001 when compared to CD uptake in the absence of inhibitor (set at 100%, unrepresented on the figure).

To investigate the internalization of cationic polyamine-based CDs by THP-1-derived macrophages, cells were incubated with 25 µg/mL of the NPs or with culture medium alone as a control for 4 h, and CD-associated fluorescence was evaluated using CLSM and FACS. CD concentration and exposure time were selected from preliminary time- and dose-dependent cytotoxicity experiments showing that the NP effects occurred rapidly and that the concentration of 25 µg/mL CDs resulted in moderate effects (Figure S1). As illustrated in Figure 3a, CDs appeared as blue fluorescent spots in the cytoplasm of treated cells (cell membranes in green), whereas such spots were absent in untreated control cells. Quantitative FACS analysis (Figure 3b) revealed a significant increase in CD-associated fluorescence in macrophages exposed to the NPs compared with controls (21-fold, *p* < 0.001), confirming the internalization of cationic polyamine-based CDs. This observation is consistent with previous work from our group [24,46]. The implication of macrophage surface receptors in CD uptake was then examined by FACS (Figure 3c). Cells were pre-treated for 1 h with receptor inhibitors and then exposed to 25 µg/mL of cationic polyamine-based CDs for 4 h. The concentrations of the inhibitors were selected based on the literature [40,42,47]. The absence of cytotoxicity of all these inhibitors at the chosen concentrations was verified (Figure S2). While the Fc receptor inhibitor Fc block and the TLR4 inhibitor Cli-095 induced a slight alteration in CD cell uptake (around 10–20% inhibition), the SR inhibitors Fucoidan, Poly-I, and Poly-G markedly inhibited CD uptake by 63%, 44%, and 32%, respectively. These data suggest that SRs, and to a lesser extent Fc receptors and TLR4, are involved in CD internalization by macrophages.

**Figure 4:**
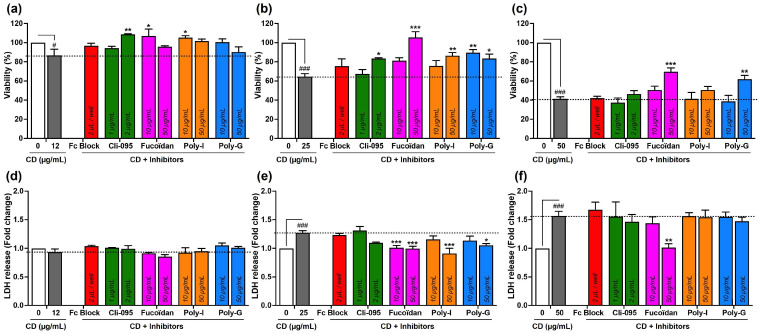

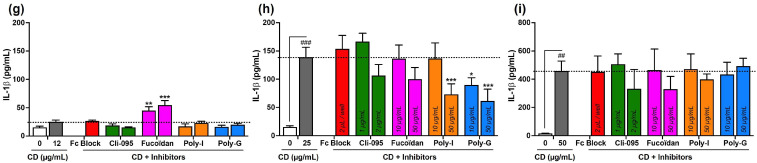
Involvement of surface receptors in CD toxicity. Cells pre-treated or not with receptor inhibitors for 1 h were exposed or not to increasing concentrations (12 (**a**,**d**,**g**), 25 (**b**,**e**,**h**), and 50 (**c**,**f**,**i**) µg/mL) of cationic polyamine-based CDs for 4 h. (**a**–**c**) Cell viability as assessed with the MTT assay. Results are expressed as the percentage of viability when compared to the control cells (not pre-treated with inhibitors and not exposed to CDs). (**d**–**f**) Cell necrosis as assessed with the LDH assay. Results are expressed in fold change in LDH release when compared to the control cells. (**g**–**i**) IL-1β secretion in cell culture supernatants as assessed by ELISA. Results are means ± SEM of *n* = 3–6 experiments. Statistical differences were determined by ANOVA followed by the Sidak’s test. ^#^ *p* < 0.05, ^##^ *p* < 0.01, and ^###^ *p* < 0.001 when compared to control cells (white bar). * *p* < 0.05, ** *p* < 0.01, and *** *p* < 0.001 when compared to the cells exposed to CDs without inhibitor pre-treatment (gray bar).

The implication of surface receptors in CD toxicity was then examined by measuring THP-1-derived macrophage viability, necrosis, and IL-1β secretion (Figure 4). These three endpoints were selected from some previous mechanistic works showing that cationic polyamine-based CDs induce loss of cell viability, lytic cell death, and activation of the NLRP3 inflammasome leading to IL-1β release in macrophages [22,23]. Together, they provide sensitive and mechanistically relevant readouts to assess the contribution of surface receptors in CD-induced macrophage responses. As before, cells were pre-treated for 1 h with receptor inhibitors and then exposed to increasing concentrations (12, 25, and 50 µg/mL) of cationic polyamine-based CDs, or to medium alone, for 4 h. The three CD concentrations were chosen to represent low, intermediate, and high toxicity conditions as determined in preliminary dose–response experiments (Figure S1). As shown in Figure 4a–c, cationic polyamine-based CDs induced a dose-dependent loss of macrophage viability (in gray). Fc Block (in red) and Cli-095 at 2 µg/mL (in green) triggered no or minimal restoration of cell viability at all CD concentrations. In contrast, inhibition of SRs by Fucoidan (in pink), Poly-I (in orange), or Poly-G (in blue) partially or completely restored cell viability when CD-induced toxicity was low (12 µg/mL) or moderate (25 µg/mL). Even under high toxicity conditions (50 µg/mL), SR inhibition maintained a protective effect, although restoration was less pronounced. The viability loss induced by cationic polyamine-based CDs resulted in a significant necrosis, which was quantified by the release of the cytoplasmic marker LDH in culture supernatants (Figure 4d–f, in gray). SR inhibition significantly reduced LDH release under moderate toxicity conditions (25 µg/mL CD), whereas its effect was negligible when CD-induced necrosis was high (50 µg/mL CD). Fc block and Cli-095 had no or little effect on the necrosis marker, regardless of CD concentration. Consistent with our previous studies [24], cationic polyamine-based CDs induced a significant increase in IL-1β secretion by macrophages (Figure 4g–i, in gray). This response was significantly attenuated by Poly-I and Poly-G under moderate toxicity conditions (25 µg/mL), while Fc Block had no effect. Fucoidan and Cli-095 also reduced IL-1β secretion, although not significantly. Under high toxicity (50 µg/mL), inhibition failed to significantly counteract the CD-induced cytokine release. Taken together, these data suggest that SRs, and to some extent TLR4, play a central role in CD-induced macrophage toxicity, particularly under low-to-moderate toxic exposure conditions.

Given the cationic nature of the CDs investigated herein, a strong involvement of TLR4 might have been expected in our study. Indeed, in the literature, TLR4 has been mainly described as recognizing cationic NPs. For example, TLR4 was reported to bind cationic liposomes [33,34] or peptide-gold NPs [35], leading to a pro-inflammatory response in THP-1-derived macrophages [33]. In addition, an interaction between graphene NPs and TLR4 has also been reported, leading to TLR4 signaling activation and subsequent oxidative stress, TNF-α secretion, and necrosis in macrophages [48]. The authors assume that the physicochemical properties of bending rigidity and stiffness of graphene would explain the interaction between graphene NP and TLR4. Our data do not exclude an interaction between cationic polyamine-based CDs and TLR4, but they indicate that it would remain very limited. By contrast, in our study, we observed a major contribution of the SRs in CD uptake and toxicity towards macrophages. In the literature, the class-A SRs were reported to be involved in the recognition and uptake of different types of NPs, such as silica [28,40,49], polystyrene [29], or gold [30,50] NPs or carbon nanotubes [31,51,52]. The structural mechanisms by which SR-A recognize such a wide range of particles and trigger their uptake remain an intriguing question. As SRs have the ability to bind polyanionic biological particles (bacteria, cells undergoing apoptosis), it has been proposed that these receptors may be involved in the recognition of negatively charged NPs [27]. By computer modeling, it has been proposed that the recognition of anionic iron oxide NPs by SR-A1 and SR-A2 receptors was mediated via the positively charged extracellular collagen-like domain of these receptors [53]. The recognition of cationic NPs by SR-A could then be indirect after the coating of the NPs by negatively charged serum proteins such as albumin [29]. To what extent serum proteins could be involved in the recognition of our cationic CDs by SR-A will be studied and discussed later in this manuscript. Besides surface charge, NP size could be a key determinant in NP recognition by SRs. Indeed, among silica NPs with sizes between 10 nm and 1 µm, NPs of 50 nm exhibited a maximal ligand effect to SR-A1 [40]. Pant et al. also investigated the role of NP size in their recognition by SR-A and showed that dendritic polyglycerol NPs of 3, 5, and 10 nm in size, corresponding to the size order of CDs, were recognized by SR-A [42]. Thus, the size of the CDs could be an element favoring their recognition by the SR-A. In addition to NP uptake, SR-A has been reported to be involved in the pro-inflammatory effects of NPs such as silica NPs [28,40]. Consistent with this, our data showed that blocking SRs reduced IL-1β secretion induced by cationic polyamine-based CDs. So, taken together, our study demonstrated the involvement of macrophage SRs in the uptake and toxicological effects of cationic polyamine-based CDs.

### 3.4. Implication of MERTK Signaling Pathway in CD Responses

**Figure 5:**
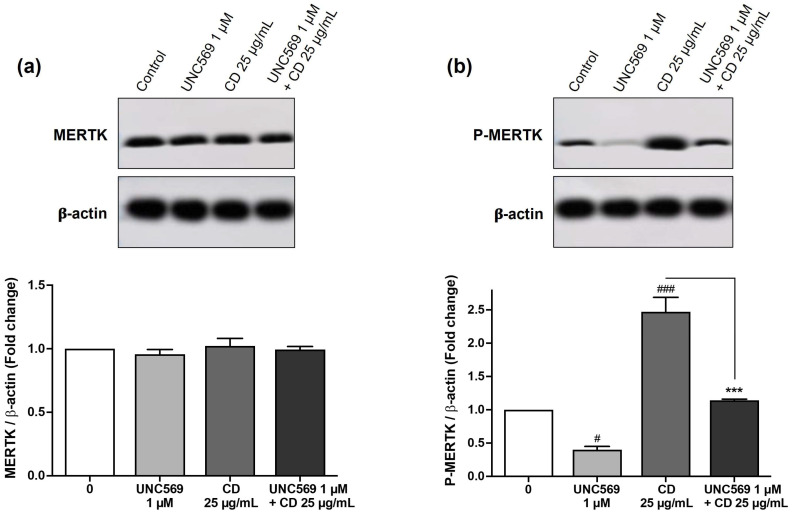
Expression (**a**) and phosphorylation (**b**) of MERTK in macrophages pre-treated or not with a MERTK phosphorylation inhibitor (UNC569, 1 µM, 1 h) and exposed or not to cationic polyamine-based CDs (25 µg/mL) for 4 h. Western blotting analysis was performed for MERTK, P-MERTK, and β-actin (*n* = 3 biological replicates). Statistical differences were determined by ANOVA followed by Sidak’s test. ^#^ *p* < 0.05 and ^###^ *p* < 0.001 when compared to the cells not exposed to the inhibitor and CDs (white bar). *** *p* < 0.001 when compared to the cells exposed to CDs without inhibitor pre-treatment (gray bar).

Among the various SRs, SR-A1 emerged as a receptor of particular interest, as it is a common target of all three SR inhibitors used in our study. Indeed, despite differences in their inhibition profiles (Fucoidan predominantly interacts with SR-A1 and SR-B1; poly-I with SR-A1 and MARCO; and poly-G with SR-A1, SR-B1, SR-B2, and MARCO), the three compounds significantly inhibited uptake and cytotoxicity of cationic polyamine-based CDs in macrophages. We therefore investigated the involvement of SR-A1 in the response evoked by our CDs in macrophages by focusing on the MERTK signaling pathway. Indeed, SR-A1 has no intracellular signaling pattern but forms complexes with MERTK, leading to MERTK phosphorylation and inflammatory signaling pathway regulation [54,55]. We evaluated the contribution of MERTK in the toxicological effects evoked by our cationic polyamine-based CDs using UNC569, an inhibitor of the MERTK phosphorylation [56,57]. Cells were pre-treated or not for 1 h with UNC569 and then exposed or not to 25 µg/mL of CDs for 4 h. As shown by Western blot, the MERTK protein was constitutively expressed (Figure 5a) and phosphorylated (Figure 5b) in THP-1-activated macrophages. As expected, cell treatment with the MERTK inhibitor UNC569 decreased MERTK phosphorylation without affecting MERTK protein levels (Figure 5a,b). Notably, cell exposure to CDs induced an increase in MERTK phosphorylation, and this process was reduced when cells were pre-treated with UNC569, demonstrating activation of the MERTK signaling pathway by cationic polyamine-based CDs (Figure 5a,b).

**Figure 6:**
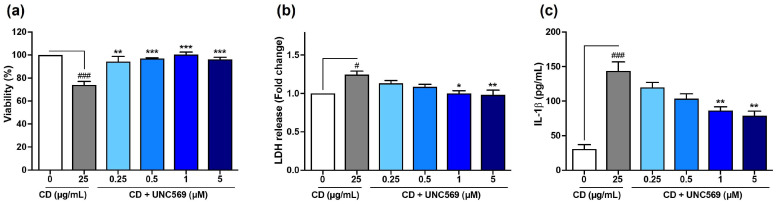
Implication of the MERTK signaling pathway in the responses evoked by cationic polyamine-based CDs in macrophages. Cells were pre-treated or not with increasing concentrations of UNC5690 (0.25, 0.5, 1, and 5 µM) for 1 h and exposed or not to 25 µg/mL of CDs for 4 h. (**a**) Cell viability as assessed with the MTT assay. Results are expressed as the percentage of viability when compared to the control cells (cells not exposed to CDs and to UNC5690). (**b**) Cell necrosis as assessed with the LDH assay. Results are expressed in fold change in LDH release when compared to the control cells. (**c**) IL-1β secretion in cell culture supernatants as assessed by ELISA. Results are means ± SEM of *n* = 3 experiments. Statistical differences were determined by ANOVA followed by the Sidak’s test. ^#^ *p* < 0.05, and ^###^ *p* < 0.001 when compared to control cells (white bar). * *p* < 0.05, ** *p* < 0.01, and *** *p* < 0.001 when compared to the cells exposed to CDs without inhibitor pre-treatment (gray bar).

Moreover, inhibition of MERTK signaling dose-dependently reduced the loss of cell viability (Figure 6a), the necrosis (Figure 6b), and the IL-1β secretion (Figure 6c) induced by CDs. Thus, taken together, our data demonstrated that the uptake of cationic polyamine-based CDs by macrophages depended on SR-A1 leading to activation of the MERTK signaling pathway, which in turn triggered the inflammatory and cytotoxic effects of the NPs. These findings are consistent with those of Nishijima et al., who reported a role for SR-A1 and MERTK in the pro-inflammatory effects of silica NPs in macrophages [40]. However, to the best of our knowledge, this is the first evidence demonstrating the involvement of SR-A1 and MERTK not only in the effects of cationic polyamine-based CDs but also more broadly in the effects of carbon-based NPs.

### 3.5. Role of Protein Corona in the Recognition of CDs by Macrophage Surface Receptors

**Figure 7:**
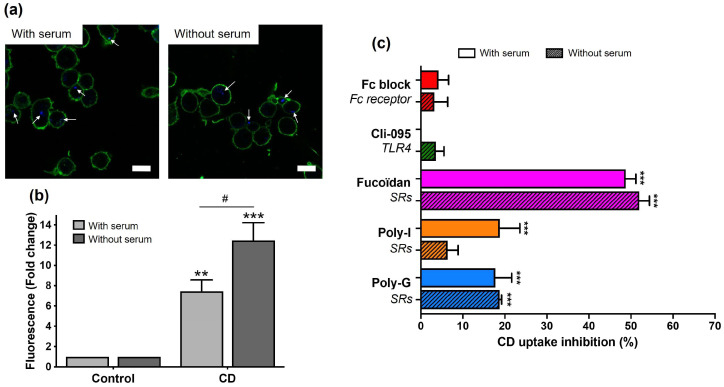
Involvement of surface receptors in CD uptake by macrophages in the presence or absence of serum. Cells were exposed to 25 µg/mL of cationic polyamine-based CDs prepared and incubated with cells in the presence or the absence of serum (or to culture medium alone with or without serum for controls), and CD uptake was assessed by CLSM and FACS. In some experiments, cells were pretreated with receptor inhibitors for 1 h before the addition of CDs. (**a**) CD uptake by cells exposed to CDs for 1 h in the presence (**left**) or the absence (**right**) of serum, assessed by CLSM analysis. Cell membranes are colored in green, and CDs appear in blue and are indicated by white arrows (scale bar = 20 µm). (**b**) Quantification of CD uptake by FACS in cells exposed to CDs for 1 h in the presence or the absence of serum. Results are expressed in fold change in fluorescence intensity when compared to control cells and are means ± SEM of *n* = 3 experiments. Statistical differences were determined by ANOVA followed by the Sidak’s test. ** *p* < 0.01 and *** *p* < 0.001 when compared to the control cells. ^#^ *p* < 0.05 when compared to the cells exposed to CDs with serum. (**c**) Involvement of surface receptors in CD uptake, as assessed by FACS. Cells were pre-treated with receptor inhibitors for 1 h before addition of CDs (25 µg/mL). The experiment was carried out in the presence or absence of serum at all steps, and internalization was measured at 1 h. Results were expressed as percent of inhibition of CD uptake with or without serum and are means ± SEM of *n* = 3 experiments. Statistical differences were determined by ANOVA followed by the Sidak’s test. *** *p* < 0.001 when compared to CD uptake with or without serum (set at 100%, unrepresented in the figure).

A remaining question was whether the protein corona had a role in the recognition of cationic polyamine-based CDs by macrophage surface receptors. To address this issue, we assessed the uptake and toxicity of the NPs prepared and incubated with macrophages in culture medium with or without serum. For the uptake study, THP-1-derived macrophages were exposed to 25 µg/mL of CDs for 1 h, and cellular uptake of CDs was assessed by monitoring CD-associated fluorescence by CLSM and FACS. As shown in Figure 7a, CDs prepared and incubated with macrophages in serum-free medium were visible in the cytoplasm of macrophages as blue spots by CLSM, similarly to CDs prepared and incubated in serum-containing culture medium. As shown in Figure 7b, FACS analysis indicated an increase in CD fluorescence signal in macrophages whether the cells were exposed to CDs in the presence or absence of serum. Remarkably, the level of CD uptake was greater when the cells were exposed to cationic polyamine-based CDs under serum-free conditions. This observation is consistent with several studies that have reported that the protein corona reduces cellular uptake of NPs [50,58], mainly because the protein corona could reduce particle adhesion to the cell membrane [59]. The role of protein corona in the recognition of cationic polyamine-based CDs by macrophage surface receptors was then assessed by measuring CD uptake by FACS (Figure 7c). Cells were pre-treated for 1 h with receptor inhibitors and then exposed to 25 µg/mL of CDs for 1 h, both protocol steps being carried out with or without serum. While Fc and TLR4 receptor inhibitors induce no or very little effects on CD uptake, the SR inhibitors (Fucoidan, Poly-I and Poly-G) decreased it significantly. However, the inhibitory effects of Fucoidan (about 50%), Poly-I (about 10–20%), and Poly-G (about 20%) were not significantly different whether the experiment was carried out with or without serum. Thus, in our study, Fc receptors appeared to play little or no role in the recognition of cationic polyamine-based CDs. One possible explanation is that the use of serum, which contains lower levels of immunoglobulins than plasma, may have limited the opsonization of cationic polyamine-based CDs and thus their interaction with Fc receptors. Consistent with this, Lunov et al. (2011) reported that Fc receptors on THP-1-derived macrophages recognized polystyrene NPs only when the particles were pre-coated with immunoglobulins [43]. In contrast, Mirshafiee et al. (2016) showed that pre-coating silica NPs with globulins did not enhance uptake of the NPs by RAW 264.7 macrophages, likely due to the presence of other serum proteins forming a steric barrier that hindered immunoglobulin-mediated binding to Fc receptors [60]. These observations highlight the complexity of Fc receptor involvement in NP recognition and suggest that further investigations are warranted.

**Figure 8:**
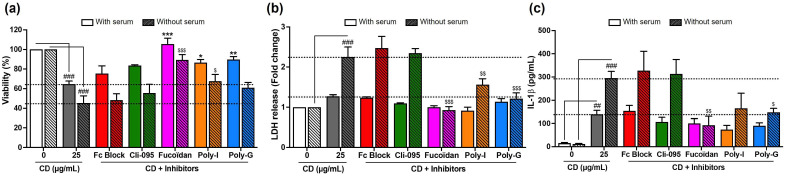
Involvement of surface receptors in the cytotoxic effects of cationic polyamine-based CDs in macrophages in the presence or absence of serum. Cells were pre-treated with receptor inhibitors for 1 h and exposed for 4 h to 25 µg/mL of CDs prepared and incubated with cells in the presence or the absence of serum (or to culture medium alone with or without serum for controls). (**a**) Cell viability as assessed with the MTT assay. Results are expressed as the percentage of viability when compared to the controls (cells not exposed to CDs). (**b**) Cell necrosis as assessed with the LDH assay. Results are expressed in fold change in LDH release when compared to the controls (cells not exposed to CDs). (**c**) IL-1β secretion in cell culture supernatants as assessed by ELISA. Results are means ± SEM of *n* = 4 experiments. Statistical differences were determined by ANOVA followed by the Sidak’s test. ^##^ *p* < 0.01, and ^###^ *p* < 0.001 when compared to the cells not exposed to CDs (white bar for control with serum and white striped bar for control without serum). * *p* < 0.05, ** *p* < 0.01 and *** *p* < 0.001 when compared to the cells exposed to CDs without inhibitor pre-treatment in the presence of serum (gray bar). ^$^ *p* < 0.05, ^$$^ *p* < 0.01, and ^$$$^ *p* < 0.001 when compared to the cells exposed to CDs without inhibitor pre-treatment in the absence of serum (gray striped bar).

The role of protein corona in the recognition of cationic polyamine-based CDs by macrophage surface receptors was next examined by measuring changes in cell viability, necrosis, and IL-1β secretion evoked by the NPs in the presence or absence of serum (Figure 8). As before, cells were pre-treated or not for 1 h with the receptor inhibitors and then exposed to 25 µg/mL of CDs for 4 h. Likely related to their higher cell uptake described above, cationic polyamine-based CDs induced greater loss of viability (Figure 8a), LDH release (Figure 8b), and IL-1β secretion (Figure 8c) in the absence of serum compared to the serum-containing condition. Nevertheless, the profiles of the effect of receptor inhibitors were broadly similar whether the experiment was performed in the presence or absence of serum. Indeed, there was no significant effect of Fc and TLR4 receptor inhibitors on CD-induced loss of viability (Figure 8a), LDH release (Figure 8b), and IL-1β secretion (Figure 8c), whether the experiments were conducted with or without serum. Likewise, blocking SRs induced a restoration of cell viability, a decrease in LDH release, and a reduction in IL-1β secretion in CD-exposed macrophages, whether the experiments were conducted with or without serum.

Taken together, our data highlighted a cellular recognition of cationic polyamine-based CDs by macrophage SRs in the absence of protein corona, which suggests a direct interaction between these NPs and SRs. Consistent with our data, Orr et al. also reported a cellular recognition of silica NPs by macrophage SR-A in serum-free culture conditions [28]. Similarly, Shannahan et al. showed that pre-treatment with an SR inhibitor decreased uptake of silver NPs with or without protein corona and reduced both cytotoxicity and the pro-inflammatory response evoked by these NPs [58]. Besides, it was reported that the interactions between SRs and gold NPs were reduced when the NPs were coated with serum proteins, whereas they were facilitated in the absence of serum proteins [50]. However, other studies have shown that some serum proteins, such as apolipoprotein APOA1 [61] or fetuin [62], would be involved in the interaction between SRs and graphene or polystyrene NPs, respectively. Undoubtedly, the involvement of protein corona in the biological effects of NPs, including their recognition mechanisms by macrophage surface receptors, is a very complex issue. Among the multiple parameters that will play a role, the physicochemical characteristics of the particles strongly influence the composition of the protein corona. With regard to CDs, our earlier studies demonstrated that the composition of the protein corona is strongly modulated by the surface charge of CDs, which in turn influences their cellular uptake and associated cytotoxicity in macrophages [38]. Specifically, cationic polyamine-based CDs with a ζ-potential above +30 mV and a surface charge density greater than 2 μmol/mg, which correspond to the CDs studied in the present work, were found to acquire a corona enriched in proteins such as vitronectin, fibulin, adiponectin, and alpha-glycoprotein. In contrast, CDs with a lower ζ-potential (+11 mV) and a reduced charge density (0.01 μmol/mg) exhibited a corona characterized by a distinct signature, notably abundant in apolipoproteins (APOA1, APOB, and APOC), albumin, and hemoglobin. It would therefore be interesting to further extend the present study to CDs with varied physicochemical characteristics in relation to their protein corona signature.

## 4. Conclusions

This study provides novel insights into the mechanisms underlying the recognition and biological effects of carbon-based NPs in macrophages. Our findings demonstrate the central role of SRs, in particular SR-A1, in macrophage uptake and toxicity of cationic polyamine-based CDs. Additionally, we identified the involvement of the MERTK signaling pathway in mediating cellular effects of these CDs, marking the first evidence of such interactions for carbon-based NPs. The protein corona appeared to have a limited impact on SR-mediated recognition of cationic polyamine-based CDs, suggesting a direct interaction between these CDs and the SRs. These results pave the way for future studies exploring how CDs might interact with the immune system. Such findings are essential for advancing the rational design of safer and more efficient NP-based therapeutic platforms.

## Figures and Tables

**Table 1:** Physicochemical characteristics of the cationic polyamine-based CDs investigated herein.

Structure	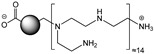
Passivation reagent	bPEI600
Hydrodynamic diameter [nm]	11.0 ± 3.4
Polydispersity index PdI	0.465 ± 0.105
ζ-potential [mV]	+31.8 ± 1.1
Surface charge density Q_ek_ [µmol/mg]	4.70
Photoluminescence λ_max_/λ_ex_/λ_em_ [nm]	350/365/460

## Data Availability

Data will be made available on request.

## Supplementary Materials

The following supporting information can be downloaded at https://www.mdpi.com/article/10.3390/toxics13090731/s1, Figure S1: Dose- and time-dependent cytotoxicity of the CDs in macrophages; Figure S2: Cytotoxicity of the pharmacological inhibitors in macrophages.

## References

[1] Himaja A.L., Karthik P.S., Singh S.P. (2015). Carbon Dots: The newest member of the carbon nanomaterials family. Chem. Rec..

[2] Truskewycz A., Yin H., Halberg N., Lai D.T.H., Ball A.S., Truong V.K., Rybicka A.M., Cole I. (2022). Carbon Dot therapeutic platforms: Administration, distribution, metabolism, excretion, toxicity, and therapeutic potential. Small.

[3] Xu X., Ray R., Gu Y., Ploehn H.J., Gearheart L., Raker K., Scrivens W.A. (2004). Electrophoretic analysis and purification of fluorescent single-walled carbon nanotube fragments. J. Am. Chem. Soc..

[4] Huang D., Zhou H., Wu Y., Wang T., Sun L., Gao P., Sun Y., Huang H., Zhou G., Hu J. (2019). Bottom-up synthesis and structural design strategy for graphene quantum dots with tunable emission to the near infrared region. Carbon.

[5] Sciortino A., Cannizzo A., Messina F. (2018). Carbon nanodots: A review—From the current understanding of the fundamental photophysics to the full control of the optical response. C.

[6] Claudel M., Fan J.H., Rapp M., Pons F., Lebeau L. (2019). Influence of carbonization conditions on luminescence and gene delivery properties of nitrogen-doped carbon dots. RSC Adv..

[7] Ghosal K., Ghosh A. (2019). Carbon dots: The next generation platform for biomedical applications. Mat. Sci. Eng. C Mater..

[8] Pierrat P., Wang R., Kereselidze D., Lux M., Didier P., Kichler A., Pons F., Lebeau L. (2015). Efficient in vitro and in vivo pulmonary delivery of nucleic acid by carbon dot-based nanocarriers. Biomaterials.

[9] Du J., Xu N., Fan J., Sun W., Peng X. (2019). Carbon dots for in vivo bioimaging and theranostics. Small.

[10] Dobrovolskaia M.A., Shurin M., Shvedova A.A. (2016). Current understanding of interactions between nanoparticles and the immune system. Toxicol. Appl. Pharmacol..

[11] Pallardy M.J., Turbica I., Biola-Vidamment A. (2017). Why the immune system should be concerned by nanomaterials?. Front. Immunol..

[12] Fadeel B. (2019). Hide and seek: Nanomaterial interactions with the immune system. Front. Immunol..

[13] Rodolpho J.M.A., Godoy K.F., Brassolatti P., Fragelli B.D.L., Camillo L., Castro C.A., Assis M., Speglich C., Longo E., Anibal F.F. (2023). Carbon black CB-EDA nanoparticles in macrophages: Changes in the oxidative stress pathway and in apoptosis signaling. Biomedicines.

[14] Kharlamova M., Kramberger C. (2023). Cytotoxicity of carbon nanotubes, graphene, fullerenes, and dots. Nanomaterials.

[15] Raja I.S., Song S.-J., Kang M.S., Lee Y.B., Kim B., Hong S.W., Jeong S.J., Lee J.-C., Han D.-W. (2019). Toxicity of zero- and one-dimensional carbon nanomaterials. Nanomaterials.

[16] Makhado B.P., Oladipo A.O., Gumbi N.N., De Kock L.A., Andraos C., Gulumian M., Nxumalo E.N. (2024). Unravelling the toxicity of carbon nanomaterials—From cellular interactions to mechanistic understanding. Toxicol. Vitr..

[17] Havrdova M., Hola K., Skopalik J., Tomankova K., Petr M., Cepe K., Polakova K., Tucek J., Bourlinos A.B., Zboril R. (2016). Toxicity of carbon dots—Effect of surface functionalization on the cell viability, reactive oxygen species generation and cell cycle. Carbon.

[18] Kuznietsova H., Géloën A., Dziubenko N., Zaderko A., Alekseev S., Lysenko V., Skryshevsky V. (2023). In vitro and in vivo toxicity of carbon dots with different chemical compositions. Discov. Nano.

[19] Singh H., Razzaghi M., Ghorbanpoor H., Ebrahimi A., Avci H., Akbari M., Hassan S. (2025). Carbon dots in drug delivery and therapeutic applications. Adv. Drug Deliv. Rev..

[20] Fan J., Claudel M., Ronzani C., Arezki Y., Lebeau L., Pons F. (2019). Physicochemical characteristics that affect carbon dot safety: Lessons from a comprehensive study on a nanoparticle library. Int. J. Pharm..

[21] Weiss M., Fan J., Claudel M., Sonntag T., Didier P., Ronzani C., Lebeau L., Pons F. (2021). Density of surface charge is a more predictive factor of the toxicity of cationic carbon nanoparticles than zeta potential. J. Nanobiotechnol..

[22] Ronzani C., Van Belle C., Didier P., Spiegelhalter C., Pierrat P., Lebeau L., Pons F. (2019). Lysosome mediates toxicological effects of polyethyleneimine-based cationic carbon dots. J. Nanopart. Res..

[23] Arezki Y., Rapp M., Lebeau L., Ronzani C., Pons F. (2022). Cationic carbon nanoparticles induce inflammasome-dependent pyroptosis in macrophages via lysosomal dysfunction. Front. Toxicol..

[24] Weiss M., Fan J., Claudel M., Lebeau L., Pons F., Ronzani C. (2021). Combined in vitro and in vivo approaches to propose a putative adverse outcome pathway for acute lung inflammation induced by nanoparticles: A study on carbon dots. Nanomaterials.

[25] Taylor P.R., Martinez-Pomares L., Stacey M., Lin H.-H., Brown G.D., Gordon S. (2005). Macrophage receptors and immune recognition. Annu. Rev. Immunol..

[26] Vita A.A., Royse E.A., Pullen N.A. (2019). Nanoparticles and danger signals: Oral delivery vehicles as potential disruptors of intestinal barrier homeostasis. J. Leukoc. Biol..

[27] Nakayama M. (2018). Macrophage recognition of crystals and nanoparticles. Front. Immunol..

[28] Orr G.A., Chrisler W.B., Cassens K.J., Tan R., Tarasevich B.J., Markillie L.M., Zangar R.C., Thrall B.D. (2011). Cellular recognition and trafficking of amorphous silica nanoparticles by macrophage scavenger receptor A. Nanotoxicology.

[29] Fleischer C.C., Payne C.K. (2014). Nanoparticle–cell interactions: Molecular structure of the protein corona and cellular outcomes. Acc. Chem. Res..

[30] França A., Aggarwal P., Barsov E.V., Kozlov S.V., Dobrovolskaia M.A., González-Fernández Á. (2011). Macrophage scavenger receptor A mediates the uptake of gold colloids by macrophages in vitro. Nanomedicine.

[31] Wang R., Lohray R., Chow E., Gangupantula P., Smith L., Draper R. (2020). Selective uptake of carboxylated multi-walled carbon nanotubes by class A type 1 scavenger receptors and impaired phagocytosis in alveolar macrophages. Nanomaterials.

[32] Tsugita M., Morimoto N., Tashiro M., Kinoshita K., Nakayama M. (2017). SR-B1 is a silica receptor that mediates canonical inflammasome activation. Cell Rep..

[33] Wilmar A., Lonez C., Vermeersch M., Andrianne M., Pérez-Morga D., Ruysschaert J.-M., Vandenbranden M., Leo O., Temmerman S.T. (2012). The cationic lipid, diC14 amidine, extends the adjuvant properties of aluminum salts through a TLR-4- and caspase-1-independent mechanism. Vaccine.

[34] Tanaka T., Legat A., Adam E., Steuve J., Gatot J., Vandenbranden M., Ulianov L., Lonez C., Ruysschaert J., Muraille E. (2008). DiC14-amidine cationic liposomes stimulate myeloid dendritic cells through toll-like receptor 4. Eur. J. Immunol..

[35] Yang H., Fung S.-Y., Xu S., Sutherland D.P., Kollmann T.R., Liu M., Turvey S.E. (2015). Amino acid-dependent attenuation of toll-like receptor signaling by peptide-gold nanoparticle hybrids. ACS Nano.

[36] Mahmoudi M., Landry M.P., Moore A., Coreas R. (2023). The protein corona from nanomedicine to environmental science. Nat. Rev. Mater..

[37] Gustafson H.H., Holt-Casper D., Grainger D.W., Ghandehari H. (2015). Nanoparticle uptake: The phagocyte problem. Nano Today.

[38] Arezki Y., Delalande F., Schaeffer-Reiss C., Cianférani S., Rapp M., Lebeau L., Pons F., Ronzani C. (2022). Surface charge influences protein corona, cell uptake and biological effects of carbon dots. Nanoscale.

[39] Chanput W., Mes J.J., Wichers H.J. (2014). THP-1 cell line: An in vitro cell model for immune modulation approach. Int. Immunopharmacol..

[40] Nishijima N., Hirai T., Misato K., Aoyama M., Kuroda E., Ishii K.J., Higashisaka K., Yoshioka Y., Tsutsumi Y. (2017). Human scavenger receptor A1-mediated inflammatory response to silica particle exposure is size specific. Front. Immunol..

[41] Alvarado-Vazquez P.A., Bernal L., Paige C.A., Grosick R.L., Moracho Vilrriales C., Ferreira D.W., Ulecia-Morón C., Romero-Sandoval E.A. (2017). Macrophage-specific nanotechnology-driven CD163 overexpression in human macrophages results in an M2 phenotype under inflammatory conditions. Immunobiology.

[42] Pant K., Pufe J., Zarschler K., Bergmann R., Steinbach J., Reimann S., Haag R., Pietzsch J., Stephan H. (2017). Surface charge and particle size determine the metabolic fate of dendritic polyglycerols. Nanoscale.

[43] Lunov O., Syrovets T., Loos C., Beil J., Delacher M., Tron K., Nienhaus G.U., Musyanovych A., Mailänder V., Landfester K. (2011). Differential uptake of functionalized polystyrene nanoparticles by human macrophages and a monocytic cell line. ACS Nano.

[44] Collot M., Ashokkumar P., Anton H., Boutant E., Faklaris O., Galli T., Mély Y., Danglot L., Klymchenko A.S. (2019). MemBright: A family of fluorescent membrane probes for advanced cellular imaging and neuroscience. Cell Chem. Biol..

[45] Forrester M.A., Wassall H.J., Hall L.S., Cao H., Wilson H.M., Barker R.N., Vickers M.A. (2018). Similarities and differences in surface receptor expression by THP-1 monocytes and differentiated macrophages polarized using seven different conditioning regimens. Cell. Immunol..

[46] Arezki Y., Cornacchia J., Rapp M., Lebeau L., Pons F., Ronzani C. (2021). A co-culture model of the human respiratory tract to discriminate the toxicological profile of cationic nanoparticles according to their surface charge density. Toxics.

[47] Jin R., Liu L., Zhu W., Li D., Yang L., Duan J., Cai Z., Nie Y., Zhang Y., Gong Q. (2019). Iron oxide nanoparticles promote macrophage autophagy and inflammatory response through activation of toll-like receptor-4 signaling. Biomaterials.

[48] Qu G., Liu S., Zhang S., Wang L., Wang X., Sun B., Yin N., Gao X., Xia T., Chen J.-J. (2013). Graphene oxide induces toll-like receptor 4 (TLR4)-dependent necrosis in macrophages. ACS Nano.

[49] Gallud A., Bondarenko O., Feliu N., Kupferschmidt N., Atluri R., Garcia-Bennett A., Fadeel B. (2017). Macrophage activation status determines the internalization of mesoporous silica particles of different sizes: Exploring the role of different pattern recognition receptors. Biomaterials.

[50] Patel P.C., Giljohann D.A., Daniel W.L., Zheng D., Prigodich A.E., Mirkin C.A. (2010). Scavenger receptors mediate cellular uptake of polyvalent oligonucleotide-functionalized gold nanoparticles. Bioconjug. Chem..

[51] Hirano S., Fujitani Y., Furuyama A., Kanno S. (2012). Macrophage receptor with collagenous structure (MARCO) is a dynamic adhesive molecule that enhances uptake of carbon nanotubes by CHO-K1 cells. Toxicol. Appl. Pharmacol..

[52] Wang R., Lee M., Kinghorn K., Hughes T., Chuckaree I., Lohray R., Chow E., Pantano P., Draper R. (2018). Quantitation of cell-associated carbon nanotubes: Selective binding and accumulation of carboxylated carbon nanotubes by macrophages. Nanotoxicology.

[53] Chao Y., Karmali P.P., Mukthavaram R., Kesari S., Kouznetsova V.L., Tsigelny I.F., Simberg D. (2013). Direct recognition of superparamagnetic nanocrystals by macrophage scavenger receptor SR-AI. ACS Nano.

[54] Todt J.C., Hu B., Curtis J.L. (2008). The scavenger receptor SR-A I/II (CD204) signals via the receptor tyrosine kinase Mertk during apoptotic cell uptake by murine macrophages. J. Leukoc. Biol..

[55] Linares-Alcántara E., Mendlovic F. (2022). Scavenger receptor A1 signaling pathways affecting macrophage functions in innate and adaptive immunity. Immunol. Investig..

[56] Koda Y., Itoh M., Tohda S. (2018). Effects of MERTK inhibitors UNC569 and UNC1062 on the growth of acute myeloid leukaemia cells. Anticancer Res..

[57] Bae S.H., Kim J.H., Park T.H., Lee K., Lee B.I., Jang H. (2022). BMS794833 inhibits macrophage efferocytosis by directly binding to MERTK and inhibiting its activity. Exp. Mol. Med..

[58] Shannahan J.H., Podila R., Aldossari A.A., Emerson H., Powell B.A., Ke P.C., Rao A.M., Brown J.M. (2015). Formation of a protein corona on silver nanoparticles mediates cellular toxicity via scavenger receptors. Toxicol. Sci..

[59] Lesniak A., Fenaroli F., Monopoli M.P., Åberg C., Dawson K.A., Salvati A. (2012). Effects of the presence or absence of a protein corona on silica nanoparticle uptake and impact on cells. ACS Nano.

[60] Mirshafiee V., Kim R., Park S., Mahmoudi M., Kraft M.L. (2016). Impact of protein pre-coating on the protein corona composition and nanoparticle cellular uptake. Biomaterials.

[61] Alnasser F., Castagnola V., Boselli L., Esquivel-Gaon M., Efeoglu E., McIntyre J., Byrne H.J., Dawson K.A. (2019). Graphene nanoflake uptake mediated by scavenger receptors. Nano Lett..

[62] Nagayama S., Ogawara K., Minato K., Fukuoka Y., Takakura Y., Hashida M., Higaki K., Kimura T. (2007). Fetuin mediates hepatic uptake of negatively charged nanoparticles via scavenger receptor. Int. J. Pharm..

