# Supplementary material for: Involvement of Surface Receptors in the Uptake and Cellular Responses Induced by Cationic Polyamine-Based Carbon Dots in Macrophages
Source: Toxics. 2025 Aug 30;13(9):731. doi: 10.3390/toxics13090731 (PMC12473518; doi:10.3390/toxics13090731)
Supplement: Supplementary file 1 [file toxics-13-00731-s001.zip › toxics-3782619-supplementary.pdf]

Supplementary Data File

Involvement of Surface Receptors in the Uptake and Cellular Responses Induced by Cationic Polyamine-based Carbon Dots in Macrophages

Agathe Cerland, Ezeddine Harmouch, Mickaël Rapp, Luc Lebeau, Françoise Pons and Carole Ronzani

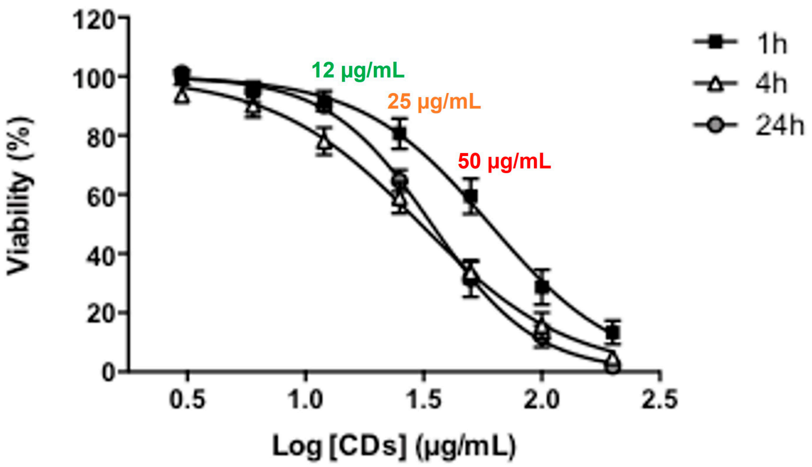

Figure S1. Dose- and time-dependent cytotoxicity of the cationic polyamine-based CDs in macrophages. Cells were incubated or not with increasing concentrations (3; 6; 12; 25; 50; 100 or 200 µg/mL) of the CDs for 1 h, 4 h or 24 h, and cell viability was assessed with the MTT assay at the end of the exposure period. Results are expressed as percentage of viability when compared to control (unexposed cells). They are means ± SEM of n = 3–4 experiments. Concentration-response curves were obtained after logarithmic transformation of the data and fit with the Hill equation.

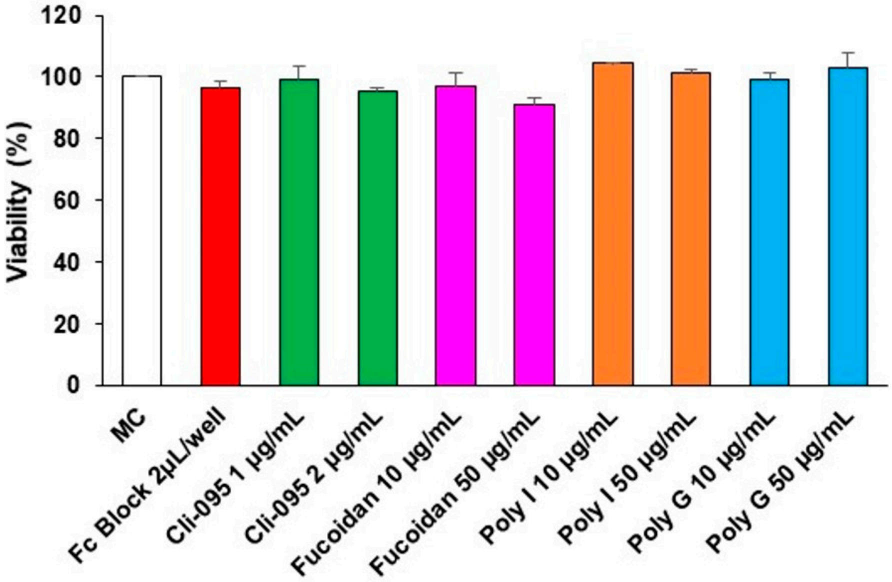

Figure S2. Cytotoxicity of the pharmacological inhibitors in macrophages. Cells were incubated or not with the inhibitors at the indicated concentrations and for 5 h to mimic the incubation conditions used in the experiments with the CDs, and cell viability was assessed with the MTT assay at the end of the exposure period. Results are expressed as percentage of viability when compared to control (unexposed cells). They are means ± SEM of n = 3 experiments.
